# The Effect of Varicocelectomy on Sperm Parameters in Subfertile Men with Clinical Varicoceles Who Have Asthenozoospermia or Teratozoospermia with Normal Sperm Density

**DOI:** 10.1155/2013/698351

**Published:** 2013-10-21

**Authors:** Basri Cakiroglu, Orhun Sinanoglu, Ramazan Gozukucuk

**Affiliations:** ^1^Department of Urology, Hisar Intercontinental Hospital, Saray Mahallesi, Siteyolu Caddesi No. 7, Umraniye, 34768 Istanbul, Turkey; ^2^Department of Urology, Maltepe University Medical School, Maltepe, 34844 Istanbul, Turkey; ^3^Department of Infectious Diseases and Clinical Microbiology, Hisar Intercontinental Hospital, Saray Mahallesi, Siteyolu Caddesi No. 7, Umraniye, 34768 Istanbul, Turkey

## Abstract

*Background*. To compare preoperative and postoperative sperm parameters such as sperm count, motility, and morphology in patients with normal sperm concentration with teratozoospermia and asthenozoospermia. *Materials and Methods*. Hundred and six patients with varicocele associated with male infertility over a 5-year period were included into the study. Pre- and postvaricocelectomy seminal fluid parameters evaluation according to the World Health Organization (WHO) criteria was performed at 4–6-month intervals. *Results*. One hundred and six patients met the criteria. The mean age of patients was 24.53 ± 8.13. The mean duration of infertility was 3.6 years (range: 1.5–6.3). Only the sperm motility of patients with normospermia showed a significant improvement postoperatively. *Conclusions*. No significant improvement in sperm morphology may be obtained in patients with clinical varicocele and preoperative normospermia.

## 1. Introduction

The incidence of infertility is 10–15% among couples trying to conceive, with male infertility contributing to nearly 50% of cases [1]. Although multiple factors may play a role in male infertility, varicocele is the most frequent finding in male infertility, with a prevalence of 19–41% of men with primary infertility and 45–81% of men with secondary infertility [1, 2]. Increased scrotal temperature, reflux of metabolites from the kidney and adrenal gland, decreased volume of blood flow, and anoxia are the supposed mechanisms [3]. Varicocele is also known as the most surgically correctable cause of male infertility, and its repair is the most commonly performed surgical procedure in order to correct male infertility [4]. Previous studies have shown abnormalities in the sperm count, motility, and morphology in varicocele patients and a significant improvement in these parameters following surgical correction [5]. The postoperative outcomes of varicocelectomy operation in patients with normal sperm count but with sperms having abnormal morphology and impaired motility have not been studied much. Therefore, in this retrospective study, we compared preoperative and postoperative sperm parameters such as sperm count, motility, and morphology in patients with normal sperm concentration showing abnormal forms and decreased motility pattern.

## 2. Materials and Methods

The study included 106 patients seeking surgical treatment for varicocele in our institution from December 2008 to December 2011. There were no documented diseases that would affect the results. A basic infertility evaluation including a detailed history and a thorough physical examination was carried out. All of the patients were married with primary infertility and varicocele (grade 1–3) having normospermia either having sperms with decreased normal forms and/or motility (progressive motility and nonprogressive motility). All the patients with varicocele were tested with color Doppler ultrasound which identified it as anechoic tubular structures that dilated on Valsalva maneuver. Volume, pH, sperm density, morphology, and motility were evaluated. The normal semen parameters according to the WHO Manual for Semen Analysis were as follows [5]: volume of semen in adult males: 1.5 mL, sperm concentration: 15 × 10^6^, sperm morphology (normal forms): 4%, progressive and nonprogressive motility (PR+NP): 40%, and progressive (PR): 32%. Subinguinal varicocelectomy was performed for all of the patients by a single urologist. Four weeks after the operation, a Doppler ultrasonography imaging was performed to confirm the improvement of varicocele by the absence of venous back flow. We followed up the patients for 3 and 6 months, and seminal analysis were performed to evaluate changes in the seminal indices. 

The data was analyzed using the SPSS 14.0 statistical software for Windows (SPSS, Chicago, IL, USA). For independent variables, we used independent sample *t*-test, and for dependent variables, paired sample *t*-test was utilized. *P* values less than 0.05 were considered statistically significant.

## 3. Results

One hundred and six patients met the criteria. The mean age of patients was 24.53 ± 8.13. The mean duration of infertility was 3.6 years (range: 1.5–6.3). The pairwise comparison of the preoperative and the postoperative sperm concentrations in all the normospermic patients did not show any significant improvements (*P* = 0.105) after varicocelectomy (Table 1). Asthenozoospermia defined as sperm poor forward motility and represented as PR+NP motile sperms was found in 92 (87%) patients preoperatively. Postoperatively, asthenospermia was found in 42 (40%) of the patients. When the sperm motility of the patients obtained postoperatively was compared with the preoperative values using a paired *t*-test, there was a significant improvement in the sperm motility overall (*P* = 0.000) (Table 1). Teratospermia was defined as normal sperm morphology of less than 4%. Forty-nine (46%) patients were found to have teratozoospermia preoperatively. Following varicocelectomy, teratospermia was seen in 40 (38%) patients. Paired comparison of the preoperative and postoperative semen analyses showed that there was no significant improvement in sperm morphology after varicocelectomy in the overall group (*P* = 0.400; Table 1).

## 4. Discussion

Testicular varicose veins cause reduction in function and number of the testicular cells which is reflected as an altered sperm parameter [6]. Surgical treatment is indicated in men with varicocele when the semen analysis shows oligospermia, asthenospermia, teratospermia, or coexistence of these abnormalities. To date, previous studies have demonstrated a beneficial effect of varicocelectomy in subfertile men with varicocele who have poor sperm quality [7]. It is also known that several patients with clinical varicoceles have isolated abnormalities such as sperm motility or morphological parameters in the semen analysis and varicocelectomy is carried out in these patients as well. However, it has not been studied much whether varicocelectomy is beneficial in such patients. In this study, we tried to examine the changes in the semen parameters after varicocelectomy among the patients who have normal sperm count associated with asthenospermia and/or teratospermia.

Evidence suggests that men with normospermic varicocele respond to varicocelectomy differently from those patients who have oligospermia preoperatively due to a different pathophysiological mechanism [8]. Two important studies evaluated the postoperative outcomes of varicocele correction in normospermic patients. In one, isolated teratospermia did not show any significant improvement following varicocelectomy; in the other, neither asthenospermia nor teratozoospermia showed improvement [7, 9]. Additionally, authors claimed that performing varicocelectomy exposes this group of patients to the risk of oligozoospermia. Our data also demonstrated that these patients with preoperative normospermia did not show significant improvement in teratozoospermia; the only benefit of surgery is at sperm motility.

The possible explanation is that the poor morphology observed in varicocele patients with normal sperm density may not be only due to the presence of varicoceles. Varicocelectomy in these patients may lead to an unknown alteration or damage to the semen physiology and subsequently morphological abnormalities. With the evidence of the present and previous studies, it may therefore be recommended that normospermic subfertile men with clinical varicoceles and poor sperm motility or morphology should undergo assisted reproductive techniques rather than surgical varicocele ligation.

There are also limitations to our study; first, it is a retrospective study; second, there is lack of data concerning pregnancy outcome.

In conclusion, normospermic subfertile men with clinical varicoceles and teratozoospermia may not show statistically significant improvement in sperm morphology following varicocelectomy. We think that varicocelectomy choice for normospermic patients with teratospermia should be at least carefully considered. Comprehensive multi-institutional studies are necessary to confirm the present findings.

## Figures and Tables

**Table 1:** Effect of varicocelectomy on semen parameters in patients with normal sperm density (>15 million/mL).

No. 106	Sperm count	Morphology %	Motility (*a*) %	Motility (*a* + *b*) %
Preoperative	59.9 ± 40.2	3.6 ± 1.6	15.4 ± 8.0	29.7 ± 10.7
Postoperative	64.7 ± 43.2	3.7 ± 1.4	20.4 ± 8.4	40.3 ± 10.7
*P* value	0.105	0.400	0.000	0.000
